# Sport-specific experience modulates perceived exertion but not enjoyment or workload in recreational 3 × 3 basketball

**DOI:** 10.3389/fphys.2025.1739427

**Published:** 2026-01-06

**Authors:** Inga Lukonaitienė, Marco Pernigoni, Audinga Kniubaitė, Rasa Kreivytė, Sigitas Kamandulis, Daniele Conte

**Affiliations:** 1 Institute of Sport Science and Innovations, Lithuanian Sports University, Kaunas, Lithuania; 2 Department of Coaching Science, Lithuanian Sports University, Kaunas, Lithuania; 3 Department of Movement, Human and Health Sciences, University of Rome “Foro Italico”, Rome, Italy

**Keywords:** accelerometry, exercise, heart rate, physical fitness, wearable electronic devices

## Abstract

This study aimed to investigate the differences of previous basketball-specific experience on perceived exertion, enjoyment, physiological, and physical responses during recreational 3 × 3 basketball in active young adults. Twenty-four healthy male participants were divided into two groups: those with basketball experience (9.6 ± 4.5 years) and those with minimal or no experience (0.7 ± 0.9 years). All participants completed a 10-min recreational 3 × 3 basketball match following official FIBA rules. Heart rate responses were monitored using Polar H10 heart rate straps to determine the percentage of maximal heart rate (%HRmax), while perceptual responses were assessed through the modified Borg RPE scale (0–10) and the Exercise Enjoyment Scale (1–7). Physical demands were captured using inertial measurement units (Catapult) and included PlayerLoad/min, accelerations, decelerations, changes of direction, and jumps. Between-group differences were analyzed using t-tests for normally distributed data or Mann–Whitney U tests otherwise. No significant differences were observed between experienced and non-experienced players for %HRmax, enjoyment, or physical activity metrics (p > 0.05). However, non-experienced participants reported significantly higher RPE values than their experienced counterparts (p = 0.005; r = 0.68, *large* effect size), indicating that prior sport-specific experience may affect the perceived difficulty of a given task, even when physiological and physical outputs are similar. In conclusion, these findings suggest that perceived exertion is more sensitive to prior sport-specific experience than physiological or physical measures, underlining the need to consider participants’ backgrounds when monitoring internal load. Overall, recreational 3 × 3 basketball remains a highly enjoyable activity across experience levels and may support adherence to long-term physical activity programs.

## 1 Introduction

Recreational team sports appear to be useful tools to improve the health status of the general population (Krustrup and Krustrup, 2018; Milanović et al., 2018). For instance, playing recreational football has been shown to elicit positive adaptations on several health-related markers, such as blood pressure, resting heart rate, fat mass, and low-density lipoprotein cholesterol compared to no-exercise controls (Milanović et al., 2018). Moreover, participation in team sport is associated with high levels of enjoyment (Conte et al., 2023; Golluceli et al., 2025) and can foster social interaction in both pair-work and group settings, with positive effects on mental and social wellbeing (Krustrup and Krustrup, 2018).

Considering recreational basketball activities such as small-sided games (SSGs), this training format has been shown to elicit high physical and physiological demands, which–according to several studies–may lead to positive health and physical fitness adaptations (Stojanović et al., 2021). Specifically, findings from an 8-week recreational basketball program performed three times per week showed significant improvements in aerobic capacity, jump performance and handgrip strength in overweight and sedentary adults (Xu et al., 2025), confirming its effectiveness for fitness enhancement. Similarly, a 3-month basketball exercise program improved flexibility, balance and lower-limb strength, while reducing blood pressure and perceived exertion in middle-aged men and women (Karatrantou et al., 2024). However, not all studies report significant improvements in every outcome, indicating that adaptations to recreational basketball may vary depending on factors such as training duration, participant characteristics, and baseline fitness. The relatively low rating of perceived exertion (RPE), despite the high cardiovascular intensities reported in recreational basketball SSGs, may reflect the game-oriented and socially interactive nature of the activity, which together can elicit positive psychological responses that support long-term exercise adherence (Stojanović et al., 2021). Collectively, these findings suggest that basketball may represent an enjoyable and effective mode of exercise for improving health and fitness across a wide range of adult populations.

In this context, understanding the variables influencing the physical, physiological and psychological demands of recreational basketball activities is fundamental. Indeed, the modification of basketball game formats can affect the load imposed on players (O’Grady et al., 2020) and, in turn, potentially lead to different adaptations in long-term programs. In this regard, a previous investigation (Stojanović et al., 2021) assessing the external and internal load imposed by recreational basketball SSGs played on half court with different number of players (1v1; 2v2 and 3v3) highlighted lower internal (blood lactate concentration and RPE) and external (total accelerations and decelerations performed at medium intensity) loads in SSGs played with a higher number of players.

Player number is often the main focus in recreational basketball. However, other factors can also shape the physiological, physical, and psychological demands of the game. For instance, possessing previous sport-specific experience might influence the demands elicited by recreational basketball activities. In line with this idea, a previous investigation comparing the load and enjoyment elicited by a 3 × 3 basketball match with a gym-based, high-intensity interval training (HIIT) session found that healthy male recreational basketball players exhibited higher %HRmax and enjoyment–as well as lower RPE scores–during 3 × 3 basketball compared to HIIT (Conte et al., 2023). As noted above, such findings may be partly driven by participants’ prior basketball experience. Possibly, participants with little or no prior sport-specific experience might lack the technical competence seen in experienced players, leading to more match interruptions and fewer high-intensity actions. This could reduce their physiological and physical load, perceived exertion, and enjoyment levels, which are fundamental factors for adherence to long-term programs (Gamero et al., 2021).

Previous research in youth educational contexts has shown that early structured sport experience is linked to greater skill proficiency and fewer errors in game-like situations, and that experienced players demonstrate more efficient external load patterns (Gamero et al., 2021). However, most existing work has focused on youth populations or on how game formats influence internal and external load, while far less is known about how individual sport-specific experience affects physiological, physical, and perceptual responses in adult recreational settings. Understanding these effects may help practitioners design more effective and appropriate training sessions that account for participants’ backgrounds and capabilities. Therefore, the aim of the present study was to assess the influence of previous basketball experience on perceived exertion, enjoyment, and physiological and physical demands during recreational 3 × 3 basketball matches.

## 2 Materials and methods

### 2.1 Participants

Twenty-four apparently healthy, male, young adults volunteered to participate in this study and were equally divided in players possessing previous basketball experience (9.6 ± 4.5 years) and without or minimal basketball experience (0.7 ± 0.9 years) (Table 1). Participants were required to be free from musculoskeletal injuries in the previous 6 months and to report no cardiovascular or metabolic diseases, and the years of basketball experience reflected participation in competitive contexts depending on individual histories. Participants also self-reported engaging in at least 150 min/week of moderate-intensity activity, or 75 min/week of vigorous activity, in line with recommendations provided by the World Health Organization (Bull et al., 2020). Before the beginning of data collection, all procedures, benefits, and risks were explained to each participant, and written informed consent was obtained. The procedures received approval by the ethics committee of the Lithuanian Sports University [approval number: BNL-TRS(B)-2022-449].

**TABLE 1:** Anthropometric and fitness characteristics of study participants.

Measures	Calculations	Experienced	Non-experienced	p	ES (Interpretation)
Age (y)	Mean ± SD	23.0 ± 2.7	21.2 ± 1.8	0.026	0.542 (Large)
	Median (IQR)	22.5 (2.5)	20.8 (2.0)		
Stature (cm)	Mean ± SD	188.4 ± 9.5	183.0 ± 3.9	0.053	0.472 (Moderate)
	Median (IQR)	190.0 (10.8)	183.0 (4.5)		
Body mass (kg)	Mean ± SD	81.7 ± 15.3	76.6 ± 9.2	0.590	0.139 (Small)
	Median (IQR)	77.6 (18.9)	78.0 (10.0)		
Fat mass (%)	Mean ± SD	10.3 ± 6.0	9.5 ± 4.0	1.000	0.007 (No effect)
	Median (IQR)	10.2 (5.5)	8.9 (5.5)		
V_IFT_ (km/h)	Mean ± SD	17.7 ± 1.3	16.5 ± 1.6	0.061	0.451 (Moderate)
	Median (IQR)	18.0 (1.9)	16.3 (1.8)		
HRmax (beats/min)	Mean ± SD	194.3 ± 6.9	198.5 ± 9.7	0.099	0.403 (Moderate)
	Median (IQR)	196.0 (5.2)	199.0 (9.5)		

All variables were analyzed with a non-parametric approach.

Abbreviations: ES, effect size; SD, standard deviation; IQR, interquartile range; V_IFT_, final running speed achieved during the 30-15 Intermittent Fitness Test; HRmax, maximal heart rate.

### 2.2 Design

**FIGURE 1:**
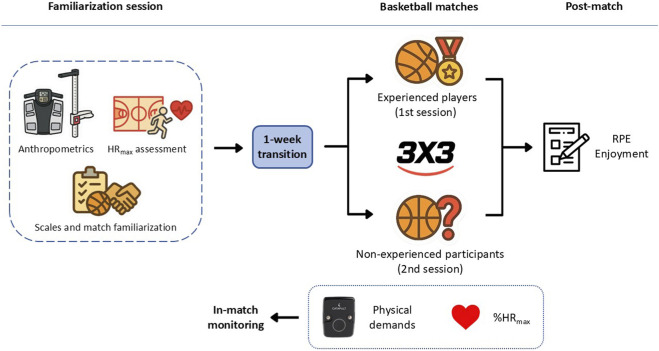
Study design and procedures.

A cross-sectional design was adopted to assess the effect of previous sport-specific experience on the physiological, physical, and perceptual demands of recreational 3 × 3 basketball matches (Figure 1). Firstly, participants took part in a familiarization session, where anthropometric characteristics (stature, body mass and %fat mass) and maximal heart rate (HRmax) were assessed. During the same session, participants also completed 4 min of recreational 3 × 3 basketball (to familiarize themselves with 3 × 3 basketball rules) and were familiarized with the scales, as well as all other study procedures. One week after the familiarization session, both experienced and non-experienced participants were first separated into two groups according to experience level, and each group of six players completed a single 3 × 3 basketball match, consisting of two teams of three players, with no repeated bouts or additional matches performed. Within each experience group, players were randomly allocated into two teams of three to simulate a real recreational scenario. It should be noted that–according to previous research (Stojanović et al., 2021) – players were allocated to teams in a randomized manner (rather than based on their skills or fitness capacity), to reproduce a real scenario commonly observed in recreational matches (particularly for non-experienced players). All matches were completed across two experimental sessions (one for experienced and one for non-experienced participants) and were carried out at a similar time of day (between 9 a.m. and 11 a.m.) to avoid any effect of circadian rhythm on the measured variables. Participants were instructed not to perform any physical activity in the 48 h preceding the experimental sessions, to maintain their regular sleeping and dietary patterns, and to avoid caffeine and alcohol on experimental day. Compliance with pre-test instructions was assessed using a custom-made questionnaire, which included basic questions regarding recent exercise participation, sleep patterns, and the consumption of caffeine and alcohol.

### 2.3 Procedures

#### 2.3.1 Anthropometrics and body composition measurements

Before the commencement of the familiarization session, participants’ anthropometric characteristics were assessed. Specifically, stature was measured without shoes using a stadiometer (Martin, GPM instrument, Siber Hegner, Zurich, Switzerland) with a precision of 0.1 cm, with the head in a standard position. Body mass and body fat were measured using an electronic body composition analyzer (TBF-300, Tanita United Kingdom Ltd., West Drayton, United Kingdom), featuring four electrodes beneath the feet. The device extrapolates overall body composition from impedance, body fat, and fat-free mass using a proprietary equation based on resistance, body mass, stature, age, and sex (Ballesteros-Pomar et al., 2012).

#### 2.3.2 Recreational 3 × 3 basketball matches

Each recreational 3 × 3 basketball match was played following the official rules of 3 × 3 basketball (https://fiba3x3.com/en/rules.html), according to the same procedures described in previous research (Conte et al., 2023). Specifically, each match was played on the half court of a regular-sized basketball court with a wooden floor, using the official size six ball for international 3 × 3 competitions. Each match lasted for a maximum of 10 min of live time, or until one of the two teams reached 21 points. Players were notified about the score in real time, and a 12-s shot clock was set, as used in official competitions. No substitutions were allowed during the match, since all teams included three players (and not 4, as in official competitions). Each match was preceded by a standardized 10-min warm-up consisting of jogging, joint mobility exercises and dynamic stretching, and ending with basketball-specific drills including dribbling and shooting.

#### 2.3.3 Perceptual measures: RPE and enjoyment

Within 5 min after each 3 × 3 match, participants’ RPE was collected using the modified CR10 scale, ranging from 0 (“rest”) to 10 (“maximal”) (Foster et al., 2001). Furthermore, the Exercise Enjoyment Scale (EES) was used to assess participants’ enjoyment, responding to the statement: “Use the following scale to indicate how much you are enjoying this exercise session” on a seven-point Likert scale (1 – “not at all”; 2 – “very little”; 3 – “slightly”; 4 – “moderately”; 5 – “quite a bit”; 6 – “very much”; 7 – “extremely”) (Stanley et al., 2009). Participants were familiarized with both scales during the familiarization session. Both scales have been previously used to assess RPE and enjoyment in recreational 3 × 3 basketball with active young adults (Stojanović et al., 2021; Conte et al., 2023).

#### 2.3.4 Physical demands

Prior to the 3 × 3 basketball matches, participants were individually equipped with inertial measurement units (IMU; Catapult ClearSky T6, Catapult Innovations, Melbourne, VIC, Australia) secured in manufacturer-provided vests between the scapulae. Match timing (live time and stoppages), PlayerLoad (PL), the number of accelerations (ACC), decelerations (DEC), changes of direction (COD), and jumps (JUMP) were collected and downloaded to the manufacturer’s proprietary software (Catapult OpenField, v1.18) for analysis. Because match durations differed across groups, all external load variables were expressed relative to live playing time (i.e., values per minute). These load measures are widely used in basketball research to quantify physical demands (Lukonaitienė et al., 2021; Russell et al., 2021; Pernigoni et al., 2025; Qarouach et al., 2025).

#### 2.3.5 Physiological demands

Participants’ HRmax was assessed during the familiarization session using the 30–15 Intermittent Fitness Test (30–15 IFT), developed for court-based intermittent sports (Buchheit, 2010) and previously used in basketball research (Conte et al., 2023; Pernigoni et al., 2024). Briefly, the test consisted of 30-s shuttle runs across a regulation basketball court, interspersed with 15-s passive recovery, with running speed dictated by incrementally faster audio signals. The test ended when participants could no longer maintain the required speed. The heart rate recorded at the final stage was considered as the HRmax, and the final running speed (V_IFT_) was also recorded. During the 3 × 3 matches, %HRmax was monitored with Polar H10 chest straps (Polar Electro Oy, Kempele, Finland) sampling at 1 Hz. If a higher heart rate was observed during match-play than during the 30–15 IFT, HRmax was updated to the highest observed value (Berkelmans et al., 2018) and %HRmax was recalculated.

### 2.4 Statistical analysis

Data are presented as mean ± standard deviation (SD) and median ± interquartile range (IQR). The Shapiro-Wilk test was used to assess data distribution for continuous dependent variables. Therefore, to identify between-group differences, an independent sample t-test was used for each normally distributed dependent variable (PL/min, DEC/min, JUMP/min, COD/min, %HRmax), while the Mann-Whitney U test was performed for non-normally distributed or violating the assumption of homogeneity of variance (age, stature, body mass, %fat mass, ACC/min, HRmax), and ordinal (V_IFT_, RPE, enjoyment) variables. For variables analyzed using a parametric approach, mean differences and their 95% confidence intervals (95% CI) were reported, along with Cohen’s d effect sizes (ES), each accompanied by their 95% CI and interpreted as: trivial, <0.20; small, 0.20–0.59; moderate, 0.60–1.19; large, 1.20–1.99; and very large, ≥2.0 (Hopkins et al., 2009). Rank-biserial correlation (r) ES were calculated to assess the magnitude of differences assessed with a non-parametric approach and results were interpreted according to Cohen’s benchmarks (small = 0.1–0.3, moderate = 0.3–0.5, and large >0.5) (Cohen, 1988). The α level was set at 0.05 and statistical analyses were performed using the Jamovi software package for Windows (version: 2.2.5; Sydney, Australia; retrieved from https://www.jamovi.org).

## 3 Results

No significant between-group differences were observed for stature, body mass, %fat mass, V_IFT_, or HRmax (all p > 0.05; Table 1). Table 2 shows differences in perceptual, physical and physiological demands. The present findings revealed no significant differences in terms of enjoyment (p = 0.059, r = 0.430, *moderate*) and %HRmax (p = 0.238, d = −0.495, *moderate*), while non-experienced participants showed higher RPE values (p = 0.005; r = 0.681, *large*) compared to experienced participants. *Trivial-to-small*, non-significant differences (p > 0.05) were found for PL/min, ACC/min, DEC/min, JUMP/min and COD/min (Table 2).

**TABLE 2:** Perceptual, physical and physiological responses to 3 × 3 match-play.

Measures	Calculations	Experienced	Non-experienced	p	ES (Interpretation)	95% CI
*RPE (AU)	Mean ± SD	5.0 ± 1.6	7.0 ± 1.2	0.005	0.681 (Large)	N/A
	Median (IQR)	5.0 (1.5)	7.0 (2.0)			
*Enjoyment (AU)	Mean ± SD	5.6 ± 1.1	6.3 ± 0.7	0.059	0.431 (Moderate)	N/A
	Median (IQR)	6.0 (1.0)	6.0 (1.0)			
PL/min (AU/min)	Mean ± SD	10.0 ± 1.5	9.9 ± 1.6	0.957	0.023 (Trivial)	−0.796; 0.840
	Median (IQR)	10.2 (2.2)	9.6 (1.5)			
*ACC/min (n/min)	Mean ± SD	1.9 ± 0.6	2.0 ± 0.7	0.975	0.015 (No effect)	N/A
	Median (IQR)	1.9 (0.5)	2.0 (0.6)			
DEC/min (n/min)	Mean ± SD	2.8 ± 0.8	2.4 ± 1.0	0.357	0.393 (Small)	−0.450; 1.218
	Median (IQR)	2.6 (1.0)	2.5 (1.0)			
JUMP/min (n/min)	Mean ± SD	2.2 ± 1.1	2.0 ± 0.9	0.609	0.217 (Small)	−0.611; 1.035
	Median (IQR)	2.2 (1.5)	1.9 (1.3)			
COD/min (n/min)	Mean ± SD	11.1 ± 2.2	11.3 ± 3.7	0.869	−0.070 (Trivial)	−0.887; 0.750
	Median (IQR)	10.2 (3.5)	10.2 (3.9)			
%HRmax	Mean ± SD	91.2 ± 3.5	89.6 ± 5.3	0.238	−0.495 (Small)	−1.311; 0.341
	Median (IQR)	91.7 (5.6)	91.8 (7.2)			

* indicates a dependent variable analyzed with a non-parametric approach; therefore, confidence intervals (CI) are not available and are reported as N/A.

Abbreviations: ES, effect size; CI, confidence interval; RPE, rating of perceived exertion; AU, arbitrary units; SD, standard deviation; IQR, interquartile range; N/A, not available; PL, PlayerLoad; ACC, accelerations; DEC, decelerations; JUMP, jumps; COD, changes of direction; %HRmax, percentage of maximal heart rate.

## 4 Discussion

The present study aimed to assess the effect of previous sport-specific basketball experience on perceived exertion, enjoyment, physiological, and physical demands during recreational 3 × 3 basketball matches. The main results indicate that–compared to participants without prior experience–those with basketball experience showed similar enjoyment, physical and physiological demands, but reported lower perceived exertion. These results highlight that previous sport-specific experience should be considered by health practitioners when monitoring training sessions, including recreational 3 × 3 basketball matches.

Our findings indicate that RPE was the only investigated variable able to differentiate between players with or without previous basketball experience. In sports science research, perceived exertion has been defined as “the conscious sensation of how hard, heavy, and strenuous a physical task is” (Marcora, 2010) and is mediated by the interaction of physiological (e.g., heart rate, lactate), performance-related (strategy, duration), and psychological (mood, motivation, exercise experience) factors (Robertson and Noble, 1997; Pageaux, 2016; Kilpatrick et al., 2020). Our findings align with this framework, and indicate that exercise experience can play an important role in shaping RPE. These mechanisms help explain why our participants differed in their perceived exertion during the 3 × 3 match.

Specifically, the higher RPE values recorded in non-experienced participants (median = 7 AU) compared to experienced participants (median = 5 AU) can be plausibly explained by task familiarity: experienced players are more accustomed to the sensations produced by 3 × 3’s typical physical load (frequent changes of direction, accelerations, decelerations, and jumps) and may better manage perceived effort. Indeed, the inexperienced participants in our study mainly practiced individual sports (e.g., track and field, swimming), which involve cyclic and linear movement patterns that differ markedly from the intermittent and multidirectional demands of 3 × 3 basketball. Changes of direction, in particular, increase neuromuscular and metabolic costs compared with straight-line locomotion (Hader et al., 2014; Silva et al., 2022; Cabarkapa et al., 2023; Sansone et al., 2023). Therefore, although the objective physiological and physical load during the 3 × 3 match was similar across groups, non-experienced participants may have perceived the session as harder than experienced participants. Another plausible contributor to the present findings is related to the participants’ fitness level. Although no statistically significant differences were detected between groups, the experienced participants displayed *moderately* higher cardiorespiratory fitness (V_IFT_) at baseline. In fact, alongside other determinants, cardiorespiratory fitness has been shown to alter perceived exertion (Haddad et al., 2017), with fitter individuals typically reporting lower RPE scores at an equivalent absolute workload (Garcin et al., 2004; Grummt et al., 2024). Overall, the present findings indicate that practitioners should interpret RPE in recreational 3 × 3 relative to players’ background and fitness, which is consistent with contemporary guidance on RPE’s validity and use in exercise participation (Kilpatrick et al., 2020), and further suggests that RPE may not fully reflect objective workload in novice participants, highlighting the importance of considering individual experience when using perceptual measures to monitor exercise intensity.

Both groups rating the 3 × 3 session as “very much” enjoyable (median = 6 on a seven-point scale), consistent with prior recreational 3 × 3 basketball work in adults (Stojanović et al., 2021; Conte et al., 2023). Crucially, enjoyment is important for long-term participation and adherence to exercise programs, as it predicts exercise habits and intentions, and can increase during the first weeks of training (Heisz et al., 2016; Teixeira et al., 2022). Accordingly, the present findings corroborate previous research (Conte et al., 2023; Golluceli et al., 2025), by confirming that recreational 3 × 3 basketball is a fun, engaging format that can help sustain adult participation in physical activity.

No between-group differences emerged for physiological or physical demands during the 3 × 3 match. While previous research in traditional basketball (Stojanović et al., 2018) suggests that higher-level players typically face greater physical demands and elevated heart rate responses than lower levels (an effect that might also be expected when comparing experienced players to non-experienced participants), this pattern was not observed in the present study. The lack of differences likely reflects structural features of the 3 × 3 format, such as the 12-s shot clock, half-court play, and (in our protocol) the absence of substitutions, which together compress the objective load and standardize responses across players (Sansone et al., 2023).

When compared to data from competitive 3 × 3 basketball, our recreational cohort recorded ∼2.0 ACC/min, ∼2.7 DEC/min and ∼1.8 JUMP/min, whereas elite matches typically involve ∼3.4 ACC/min, ∼4.4 DEC/min and ∼2.4 JUMP/min [i.e., ∼34 ACC, ∼44 DEC, and ∼24 jumps across 10-min games (Sansone et al., 2023)], reflecting modestly lower frequencies of high-intensity actions in recreational play. In contrast, COD/min in our cohort (∼11.3) sat at the upper end of the ranges reported in elite competition [∼6–9 (Sansone et al., 2023)], suggesting that the smaller court area and uninterrupted play in our format may have increased movement density. Despite these external load differences, recreational 3 × 3 still elicited vigorous internal load. Specifically, in our cohort, average %HRmax across all participants was ∼91%, which falls within the vigorous domain [according to ACSM guidelines (Ozemek et al., 2025)], and is in line with prior competitive (Sansone et al., 2023) and recreational (Conte et al., 2023) 3 × 3 reports. Similar heart rate responses have been observed during basketball SSGs (Stojanović et al., 2021) and in other team sports such as handball (Póvoas et al., 2017), reinforcing the notion that short, game-based formats can impose substantial cardiovascular demands in adults. Moreover, evidence from small-sided team handball (Hornstrup et al., 2020) suggests that prior sport experience may modulate training adaptations over time (e.g., greater fitness and body-composition improvements in novices compared to experienced participants), indicating an interesting avenue for future longitudinal research in recreational 3 × 3, with participants stratified by prior experience.

### 4.1 Limitations and future directions

This investigation examined the acute responses to a single bout of recreational 3 × 3 basketball using a convenience sample recruited from one specific setting. Considering the cross-sectional nature of the present study, we cannot infer adherence or longer-term health adaptations. Therefore, future trials should include larger and more diverse samples and employ longitudinal designs to determine whether the acute patterns observed here translate to differential adherence and health benefits across experience levels. Moreover, the sample consisted exclusively of men and was relatively small, which may limit generalizability and statistical power. Future research should include larger and more diverse samples, including women, to strengthen external validity. In addition, although all participants met WHO recommendations for weekly physical activity, their physical activity backgrounds varied, which may have influenced perceptual responses. Future studies should consider accounting for habitual activity patterns when examining the effects of sport-specific experience. Furthermore, our sample mainly included active adults from Lithuania–a country where basketball holds strong cultural significance–which likely enhanced its appeal among participants. Hence, future studies should examine whether similarly high enjoyment is observed in contexts where basketball is less culturally prominent, or in other recreational sports.

## 5 Conclusion and practical applications

The present study demonstrates that previous basketball experience results in subjective perceptions of exertion that are different during recreational 3 × 3 basketball, without affecting objective physical or physiological responses. These findings suggest that perceptual responses–particularly RPE–are sensitive to prior sport exposure even when actual demands remain consistent. For health professionals and sport practitioners, this highlights the importance of accounting for individual background when interpreting internal-load measures or designing group-based physical-activity programs. Overall, recreational 3 × 3 basketball remains a highly enjoyable and physically engaging activity across experience levels and may serve as a valuable tool for promoting exercise adherence in diverse populations, supporting its inclusion in community-based and public health physical activity initiatives.

## Data Availability

The raw data supporting the conclusions of this article will be made available by the authors, without undue reservation.

## References

[B1] Ballesteros-Pomar M. D. Calleja-Fernández A. Diez-Rodríguez R. Vidal-Casariego A. Blanco-Suárez M. D. Cano-Rodríguez I. (2012). Comparison of different body composition measurements in severely obese patients in the clinical setting. Nutr. Hosp. 27, 1626–1630. 10.3305/NH.2012.27.5.5989 23478715

[B2] Berkelmans D. M. Dalbo V. J. Fox J. L. Stanton R. Kean C. O. Giamarelos K. E. (2018). Influence of different methods to determine maximum heart rate on training load outcomes in basketball players. J. Strength Cond. Res. 32, 3177–3185. 10.1519/JSC.0000000000002291 30540282

[B3] Buchheit M. (2010). The 30-15 intermittent fitness test: 10 year review. Myorobie J. 1, 278.

[B4] Bull F. C. Al-Ansari S. S. Biddle S. Borodulin K. Buman M. P. Cardon G. (2020). World Health Organization 2020 guidelines on physical activity and sedentary behaviour. Br. J. Sports Med. 54, 1451–1462. 10.1136/BJSPORTS-2020-102955 33239350 PMC7719906

[B5] Cabarkapa D. Krsman D. Cabarkapa D. V. Philipp N. M. Fry A. C. (2023). Physical and performance characteristics of 3×3 professional male basketball players. Sports 11, 17. 10.3390/SPORTS11010017 36668721 PMC9863738

[B6] Cohen J. (1988). Statistical power analysis for the behavioral sciences. 2nd ed. New York, NY: Lawrence Erlbaum Associates.

[B7] Conte D. Lukonaitiene I. Matulaitis K. Snieckus A. Kniubaite A. Kreivyte R. (2023). Recreational 3×3 basketball elicits higher heart rate, enjoyment, and physical activity intensities but lower blood lactate and perceived exertion compared to HIIT in active young adults. Biol. Sport 40, 889–898. 10.5114/BIOLSPORT.2023.122478 37398970 PMC10286609

[B8] Foster C. Florhaug J. A. Franklin J. Gottschall L. Hrovatin L. A. Parker S. (2001). A new approach to monitoring exercise training. J. Strength Cond. Res. 15, 109–115. 10.1519/00124278-200102000-00019 11708692

[B9] Gamero M. G. García-Ceberino J. M. Ibáñez S. J. Feu S. (2021). Influence of the pedagogical model and experience on the internal and external task load in school basketball. Int. J. Environ. Res. Public Health 18, 11854. 10.3390/ijerph182211854 34831609 PMC8623569

[B10] Garcin M. Mille-Hamard L. Billat V. (2004). Influence of aerobic fitness level on measured and estimated perceived exertion during exhausting runs. Int. J. Sports Med. 25, 270–277. 10.1055/S-2004-819939 15162246

[B11] Golluceli B. Kamandulis S. Pernigoni M. Lukonaitiene I. Kreivyte R. Conte D. (2025). Physiological, perceived, and physical demands of recreational 3×3 basketball and high-intensity interval training in sedentary adult women. Biol. Sport 42, 89–96. 10.5114/BIOLSPORT.2025.148547 41048231 PMC12490309

[B12] Grummt M. Hafermann L. Claussen L. Herrmann C. Wolfarth B. (2024). Rating of perceived exertion: a large cross-sectional study defining intensity levels for individual physical activity recommendations. Sport. Med. - Open 10, 71. 10.1186/S40798-024-00729-1 38856875 PMC11164849

[B13] Haddad M. Stylianides G. Djaoui L. Dellal A. Chamari K. (2017). Session-RPE method for training load monitoring: validity, ecological usefulness, and influencing factors. Front. Neurosci. 11, 612. 10.3389/FNINS.2017.00612/PDF 29163016 PMC5673663

[B14] Hader K. Mendez-Villanueva A. Ahmaidi S. Williams B. K. Buchheit M. (2014). Changes of direction during high-intensity intermittent runs: neuromuscular and metabolic responses. BMC Sports Sci. Med. Rehabil. 6, 2. 10.1186/2052-1847-6-2 24417863 PMC3904414

[B15] Heisz J. J. Tejada M. G. M. Paolucci E. M. Muir C. (2016). Enjoyment for high-intensity interval exercise increases during the first six weeks of training: implications for promoting exercise adherence in sedentary adults. PLoS One 11, e0168534. 10.1371/JOURNAL.PONE.0168534 27973594 PMC5156428

[B16] Hopkins W. G. Marshall S. W. Batterham A. M. Hanin J. (2009). Progressive statistics for studies in sports medicine and exercise science. Med. Sci. Sports Exerc. 41, 3–13. 10.1249/MSS.0b013e31818cb278 19092709

[B17] Hornstrup T. Póvoas S. Helge J. W. Melcher P. S. Fristrup B. Andersen J. L. (2020). Cardiovascular and metabolic health effects of team handball training in overweight women: impact of prior experience. Scand. J. Med. Sci. Sport. 30, 281–294. 10.1111/sms.13563 31596971

[B18] Karatrantou K. Pappas K. Batatolis C. Ioakimidis P. Gerodimos V. (2024). A 3-month modified basketball exercise program as a health-enhancing sport activity for middle-aged individuals. Life 14, 709. 10.3390/LIFE14060709 38929692 PMC11205037

[B19] Kilpatrick M. Newsome A. Foster C. Robertson R. Green M. (2020). Scientific rationale for RPE use in fitness assessment and exercise participation. ACSMs. Health Fit. J. 24, 24–30. 10.1249/FIT.0000000000000587

[B20] Krustrup P. Krustrup B. R. (2018). Football is medicine: it is time for patients to play. Br. J. Sports Med. 52, 1412–1413. 10.1136/BJSPORTS-2018-099377 29886433 PMC6241624

[B21] Lukonaitienė I. Conte D. Paulauskas H. Pliauga V. Kreivyte R. Stanislovaitiene J. (2021). Investigation of readiness and perceived workload in junior female basketball players during a congested match schedule. Biol. Sport 38, 341–349. 10.5114/BIOLSPORT.2021.99702 34475617 PMC8329967

[B22] Marcora S. M. (2010). “Effort: perception of,” in Encyclopedia of perception. Editor Goldstein E. B. (Thousand Oaks, CA: SAGE Publications Inc.), 380–383.

[B23] Milanović Z. Pantelić S. Čović N. Sporiš G. Mohr M. Krustrup P. (2018). Broad-spectrum physical fitness benefits of recreational football: a systematic review and meta-analysis. Br. J. Sports Med. 53, 926–939. 10.1136/BJSPORTS-2017-097885 29371223 PMC6662951

[B24] Ozemek C. Bonikowske A. Christle J. Gallo P. (2025). ACSM’s guidelines for exercise testing and prescription. 12th ed. Lippincott Williams & Wilkins.

[B25] O’Grady C. J. Fox J. L. Dalbo V. J. Scanlan A. T. (2020). A systematic review of the external and internal workloads experienced during games-based drills in basketball players. Int. J. Sports Physiol. Perform. 15, 603–616. 10.1123/ijspp.2019-0785 32294618

[B26] Pageaux B. (2016). Perception of effort in exercise science: definition, measurement and perspectives. Eur. J. Sport Sci. 16, 885–894. 10.1080/17461391.2016.1188992 27240002

[B27] Pernigoni M. Calleja-González J. Lukonaitienė I. Tessitore A. Stanislovaitienė J. Kamarauskas P. (2024). Comparative effectiveness of active recovery and static stretching during post-exercise recovery in elite youth basketball. Res. Q. Exerc. Sport 95, 272–280. 10.1080/02701367.2023.2195457 37039750

[B28] Pernigoni M. Cesanelli L. Šimkus L. Shah H. Gorbas J. Coletta F. (2025). Boost or bust? A randomized crossover study on pre-exercise caffeine supplementation for fatigue management in basketball. Nutrition 139, 112855. 10.1016/J.NUT.2025.112855 40543159

[B29] Póvoas S. C. A. Castagna C. Resende C. Coelho E. F. Silva P. Santos R. (2017). Physical and physiological demands of recreational team handball for adult untrained men. Biomed. Res. Int. 2017, 6204603. 10.1155/2017/6204603 28466014 PMC5390542

[B30] Qarouach A. Conte D. Sansone P. Pernigoni M. (2025). A dual-tech approach to measuring defensive physical demands in basketball pick-and-rolls during official games: inertial sensors and video analysis. Appl. Sci. 15, 3860. 10.3390/APP15073860

[B31] Robertson R. J. Noble B. J. (1997). Perception of physical exertion: methods, mediators, and applications. Exerc. Sport Sci. Rev. 25, 407–452. 10.1249/00003677-199700250-00017 9213100

[B32] Russell J. L. McLean B. D. Impellizzeri F. M. Strack D. S. Coutts A. J. (2021). Measuring physical demands in basketball: an explorative systematic review of practices. Sport. Med. 51, 81–112. 10.1007/s40279-020-01375-9 33151481

[B33] Sansone P. Conte D. Tessitore A. Rampinini E. Ferioli D. (2023). A systematic review on the physical, physiological, perceptual, and technical-tactical demands of official 3 × 3 basketball games. Int. J. Sports Physiol. Perform. 18, 1233–1245. 10.1123/IJSPP.2023-0104 37567576

[B34] Silva A. F. González-Fernández F. T. Aquino R. Akyildiz Z. Vieira L. P. Yıldız M. (2022). Analyzing the within and between players variability of heart rate and locomotor responses in small-sided soccer games performed repeatedly over a week. Healthcare 10, 1412. 10.3390/HEALTHCARE10081412 36011069 PMC9408770

[B35] Stanley D. Williams S. Cumming J. (2009). Preliminary validation of a single-item measure of exercise enjoyment: the exercise enjoyment scale. J. Sport Exerc. Psychol. 31, S138–S139.

[B36] Stojanović E. Stojiljković N. Scanlan A. Dalbo V. Berkelmans D. Milanović Z. (2018). The activity demands and physiological responses encountered during basketball match-play: a systematic review. Sport. Med. 48, 111–135. 10.1007/s40279-017-0794-z 29039018

[B37] Stojanović E. Stojiljković N. Stanković R. Scanlan A. T. Dalbo V. J. Milanović Z. (2021). Recreational basketball small-sided games elicit high-intensity exercise with low perceptual demand. J. Strength Cond. Res. 35, 3151–3157. 10.1519/JSC.0000000000003306 31403572

[B38] Teixeira D. S. Rodrigues F. Cid L. Monteiro D. (2022). Enjoyment as a predictor of exercise habit, intention to continue exercising, and exercise frequency: the intensity traits discrepancy moderation role. Front. Psychol. 13, 780059. 10.3389/FPSYG.2022.780059/PDF 35250719 PMC8894246

[B39] Xu Q. Silva R. M. Zmijewski P. Li T. Y. Li J. Y. Yang L. X. (2025). Enhancing physical fitness using recreational soccer and basketball: a parallel-controlled 8-week study involving overweight and obese individuals, with consideration of sex-related interactions. Biol. Sport 42, 47–58. 10.5114/BIOLSPORT.2025.139081 39758168 PMC11694193

